# Stem Cell-Based Disease Modeling and Cell Therapy

**DOI:** 10.3390/cells9102193

**Published:** 2020-09-29

**Authors:** Xiaowen Bai

**Affiliations:** Department of Cell Biology, Neurobiology & Anatomy, Medical College of Wisconsin, Milwaukee, WI 53226, USA; xibai@mcw.edu; Tel.: +1-(414)-955-5755; Fax: +1-(414)-955-6517

**Keywords:** stem cells, induced pluripotent stem cells, cancer stem cells, organoids, cardiomyocytes, maturation, CRISPR/Cas9, neurodegeneration, cell therapy

## Abstract

Stem cell science is among the fastest moving fields in biology, with many highly promising directions for translatability. To centralize and contextualize some of the latest developments, this Special Issue presents state-of-the-art research of adult stem cells, induced pluripotent stem cells (iPSCs), and embryonic stem cells as well as cancer stem cells. The studies we include describe efficient differentiation protocols of generation of chondrocytes, adipocytes, and neurons, maturation of iPSC-derived cardiomyocytes and neurons, dynamic characterization of iPSC-derived 3D cerebral organoids, CRISPR/Cas9 genome editing, and non-viral minicircle vector-based gene modification of stem cells. Different applications of stem cells in disease modeling are described as well. This volume also highlights the most recent developments and applications of stem cells in basic science research and disease treatments.

## 1. Introduction

Stem cells are present in the human body at all stages of life, from the earliest times of development (e.g., embryonic stem cells (ESCs) and fetal stem cells) through adulthood (various adult stem cells) [1,2,3]. Different types of stem cells differ in their proliferation and differentiation capacity, and cell sources, which results in their various potential applications in cell therapy and disease modeling. Notably, adult stem cells, ESCs, induced pluripotent stem cells (iPSCs), and cancer stem cells (CSCs) are widely used in basic science research and clinical application. The primary functions of adult stem cells, such as adipose tissue-derived stem cells (ADSCs), are to maintain cell homeostasis in tissues. They can replace cells that die due to injury or disease. Adult stem cells have limited proliferation and differentiation potential in comparison with ESCs and iPSCs. ESCs are derived from inner mass cells of the blastocyst-stage of mammalian embryo that are three to five days old. They can self-renew indefinitely and differentiate into cell types of all three germ layers [4,5,6]. iPSCs are artificial pluripotent stem cells and can be reprogrammed from many somatic cells such as skin and blood cells. iPSCs are similar to ESCs in the capacity of proliferation and differentiation [7,8,9]. Cancer stem cells are tumor-initiating clonogenic cells. It is widely assumed that cancer stem cells may arise from normal stem cells that undergo gene mutations via complex mechanisms. Cancer stem cells play important roles in cancer growth, metastasis, and recurrence. Therefore, targeting cancer stem cells could provide a promising way to treat various types of solid tumors [10,11].

Regenerative cell therapy has the potential to heal or replace tissues and organs damaged by age, disease, or injury. Stem cells represent a great promise as a cell source for regenerative cell therapy and have received increasing attention from basic scientists, clinicians, and the public. A rapidly growing host of clinical applications of these stem cells are being developed. Adult stem cells can be used for patients’ own cells and there are no controversial issues in the aspects of immunorejection, ethics, and tumorigenesis. Thus, they are distinctly advantaged as being acceptable to all patients and widely used in clinical trials [3,12,13,14]. The therapeutic effect and safe use of ESCs and iPSCs are increasingly validated in the treatment of multiple diseases such as myocardial infarction, spinal cord injury, and macular degeneration [15,16,17,18,19,20]. In addition to being useful tools for treating disease, stem cells are useful tools for learning about disease as well. In particular, recent progress in the field of iPSCs has opened the doors to a new era of disease modelling. iPSCs can be generated from diverse patient populations, expanded, and differentiated into a disease-related specific cell types (e.g., neurons and cardiomyocytes) that can be either cultured as two-dimensional (2D) monolayers or included in stem cell-derived organoids, which can then be used as a tool to improve the understanding of disease mechanisms and to test therapeutic interventions [9,21,22]. 

This Special Issue includes both research [23,24,25,26,27,28,29,30,31,32] and reviews articles [10,33,34,35,36,37,38,39,40,41,42] which cover wide ranges of stem cell research: adult stem cells, cancer stem cells, pluripotent stem cells, and complex 3D organoid/cell aggregate models [26,27,33], with the focuses on stem cell biology/technology [10,23,24,25,26,31,32,34], and stem cell-based disease modeling [10,27,29,31,33,38,43] and cell therapy [24,28,30,32,35,36,37,39,40,41]. 

## 2. Stem Cell Biology and Technology 

Generation of sufficient, safe, and functional stem cells or stem cell-derived cells/organoids by an efficient, but simple and rapid differentiation method is important for their effective application in disease modeling and cell therapy. The following articles describe the generation of MSCs, chondrocytes, neurons, more matured cardiomyocytes (CMs), and 3D cerebral organoids from iPSCs as well as the use of CRISPR/Cas9 technology for gene editing on stem cells. 

MSCs have been demonstrated to be a promising option for cellular therapies given their curative properties of immunomodulation, trophic support and homing, and differentiation into specific cells of a damaged tissue, as well as their poor immunogenicity allowing allogenic transplantation without strong immunosuppressants [44,45]. Karam et al. developed a simple and highly efficient all-trans-retinoic acid-based method for generating an off-the-shelf and scalable number of iPSC-derived MSCs with enhanced adipogenic potential [23].

Human degenerative cartilage has low regenerative potential due to the poor proliferation of chondrocytes. Rim et al. developed a protocol of generating chondrocytes from human iPSCs using non-viral minicircle vectors containing bone morphogenetic protein 2 (BMP2) and transforming growth factor beta 3 (TGFβ3). Minicircles are vectors with eliminated bacterial backbones and transcription units. iPSC-derived MSC pellets transfected with minicircle vectors encoding BMP2 and TGFβ3 differentiated into chondrocytes. The implanted minicircle-based chondrogenic pellets recovered the osteochondral defects in rat models. This work is a proof-of-concept study that describes the potential application of minicircle vectors in cartilage regeneration using iPSCs [24]. 

Ishikawa et al. tested the effect of adding microRNAs, miRNA-9/9*, miR-124 (miR-9/9*-124), and Bcl-xL genes (BmiRs) for the neuronal induction of human iPSCs using Tet-On-driven expression of the neurogenin2 gene (Ngn2). They demonstrated that the addition of BmiRs to the Ngn2 expression system in iPSCs enhanced maturation of the differentiating neuronal cells. Moreover, when applying this method to iPSCs from Alzheimer’s disease (AD) patients, cellular phenotypes such as increased amount of extracellular secretion of amyloid β42 was observed in a shorter culture period than those previously reported. Thus, the induction method combining Ngn2 and miR-9/9*-124 might enable more rapid and simple screening for various types of neuronal disease phenotypes and promote drug discovery [31]. 

iPSC-derived CMs (iPSC-CMs) are a promising cell source for myocardial regeneration, disease modeling and drug assessment. However, iPSC-CMs typically exhibit fetal CM-like characteristics that are different from adult CMs in several aspects such as cellular structure and metabolism. The immaturity of iPSC-CMs produced by currently widely used protocols thereby limits their applications. To address this barrier, Horikoshi et al. took advantage of the adult CMs’ unique major energy substrate utilization property to mature iPSC-CMs by culturing them in fatty acid-contained maturation medium. The results showed that fatty acid-contained medium enhanced iPSC-CM maturation in several aspects such as cell morphology, structure, gene and protein expression, and metabolism. These matured highly purified iPSC-CMs will serve as a valuable model that allows the study of metabolic changes mediated by cardiac diseases. 

The development of 3D cerebral organoid technology using human iPSCs provides a promising platform to study how brain diseases are appropriately modeled and treated. So far, understanding of the characteristics of organoids is still in its infancy due to the novelty of the cerebral organoid field. Logan and coworkers’ studies highlight dynamic development and cellular heterogeneity, the presence of functional channel activities, and electrophysiological drug response of organoids. The findings help us better understand this cerebral organoid-based cutting-edge platform and its wide uses in modeling human brains in the states of health and disease, and testing drug toxicity and therapeutic efficacy [26]. 

Recent advances in genome engineering based on the CRISPR/Cas9 technology have revolutionized our ability to manipulate genomic DNA. Its use in human iPSCs has allowed a wide range of mutant iPSC lines to be obtained at an unprecedented rate. The combination of these two groundbreaking technologies has tremendous potential, from disease modeling to stem cell-based therapies. Mianne described a pipeline of strategies to efficiently generate, sub-clone, and characterize CRISPR/Cas9-edited iPSC lines in the function of the introduced mutation (indels, point mutations, insertion of large constructs, and deletions) [34]. 

## 3. Stem Cell-Based Therapy

Stem cell-based therapies are defined as any treatment for a disease or a medical condition that fundamentally involves the use of any type of viable human stem cells including ESCs, iPSCs, and adult stem cells for autologous and allogeneic therapies. Emerging evidence of stem cell-based therapeutic effects from preclinical and clinical studies suggest that regenerative strategies may one day become a treatment for a wide range of vexing diseases [46,47,48,49]. The current progress of therapies using various stem cells such as MSCs (e.g., ADSCs and BM-MSCs), urine-derived stem cells, iPSCs, and spermatogonial stem cells (SSCs) are described and discussed in this Special Issue. Various mechanisms (e.g., paracrine protection, cell replacement, and gene modification) of underlying stem cell-based therapeutic effect are discussed as well as described below.

MSCs are easily extracted from bone marrow, fat, skin, and synovium, and differentiate into various cell lineages according to the requirements of specific biomedical applications. In recent decades, the biomedical applications of MSCs have attracted increasing attention. Han et al. present comprehensive overview of MSC isolation and differentiation methods, alongside their preclinical and clinical applications in regenerative medicine. Challenges for MSC-based therapies are also discussed. For instance, different culture conditions may affect cell proliferation and differentiation potential. Despite the current challenges, MSC-based tissue engineering represents a promising clinical strategy in the field of regenerative medicine [41]. Jacob et al. summarize the pre-clinical and clinical studies using MSCs and tissue engineering for meniscal repair and regeneration. Available literature demonstrates that MSCs appear to be safe and effective in meniscal repairs [40].

Alt et al. provide a comprehensive and translational understanding of the potential of UA-ADRCs (Uncultured Autologous Adipose Derived Regenerative Cells) and their application in regenerative medicine. The authors explain how UA-ADRCs are used in regenerative medicine, how these cells are characterized, the rationale and advantages of using UA-ADRCs in regenerative medicine, and how UA-ADRCs exert their functions in tissue regeneration. They also provide profound basic and clinical evidence demonstrating that tissue regeneration with UA-ADRCs is safe and effective. Most importantly, UA-ADRCs have the physiological capacity to adequately regenerate tissue for a broad range of clinical applications. This is in striking contrast to embryonic cells, as the adult stem cells strictly depend on signaling from the microenvironment. UA-ADRCs are relatively safe as well, as there are no cases of teratoma shown in the literature or clinical studies with adult stem cells [35].

MSCs can sense the microenvironment of the injury site and secrete various paracrine factors and extracellular vesicles (EVs) that serve several reparative functions, including antiapoptotic, anti-inflammatory, antioxidative, antifibrotic, and/or antibacterial effects in response to environmental cues to enhance regeneration of the damaged tissue. Therefore, the therapeutic efficacy of MSCs might be dependent on their paracrine potency [2,50,51,52]. Stem cell-derived secretome represents one promising therapeutic strategy. Teixeira et al. found that BM-MSCs secretome resulted in a significant amelioration in the animal behavior in a Parkinson’s Disease rat model [30]. EVs are membrane-bound cellular products (30–1000 nm) that contain various classes of nucleic acids as well as soluble and transmembrane proteins. EVs play important roles in intercellular communications, immune modulation, senescence, proliferation, differentiation, and maintaining tissue homeostasis. Marzano et al. showed that treatment of cortical spheroids with human iPSC-secreted EVs exhibited neural protective abilities in Aβ42 oligomer-treated cultures, suggesting that EVs secreted by iPSCs can be possible therapeutic options for treating neural degeneration [32].

Gene modified stem cells therapy is useful for certain genetic disorders. Stem cells can be used to delivery artificially produced molecules for therapy application. Human artificial chromosomes (HACs), including the de novo synthesized alphoid^tetO^-HAC, are a powerful tool for introducing genes of interest into eukaryotic cells. HACs are mitotically stable, non-integrative episomal units that have a large transgene insertion capacity, and allow efficient and stable transgene expression. Ponomartsev et al. found that the alphoid^tetO^-HAC vector does not interfere with the pluripotent state and provides stable transgene expression in human iPSCs and mouse ESCs. They used HAC/iPSC-based strategies for the treatment of hemophilia A monogenic disease. This study is the first step towards treatment development for hemophilia A monogenic disease with the use of a new generation of the synthetic chromosome vector—the alphoid^tetO^-HAC [28].

SSCs are the only adult stem cells that are capable of passing genes onto the next generation. Ibtisham et al. summarize the progress made in offering efficient methods for SSC isolation, characterization, in vitro 2D and 3D suspension culture systems, and in vitro differentiation, particularly using primate models. The authors also review some of the salient proposed approaches for the preservation and restoration of fertility in prepubertal and pubertal patients using currently-available and potential future SSC-driven biotechnological strategies [36]. 

Several congenital and acquired pathologies can affect human urinary tract, such as hypospadias, strictures, fistulas, trauma, and cancer. Significant advancements in the isolation and utilization of urine-derived stem cells have provided opportunities for this less invasive, limitless, and versatile source of cells to be employed in urologic tissue-engineered replacement. Abbas et al. discuss the double-edged sword of using urine stem cell-based urological tissue engineering for the treatment of urinary injury. Urine-derived stem cells have a high potential to differentiate into urothelial and smooth muscle cells. However, urinary tract reconstruction via tissue engineering is peculiar as it takes place in a milieu of urine that imposes certain risks on the implanted cells and scaffolds as a result of the highly cytotoxic nature of urine. This concern should be considered thoughtfully when designing a suitable approach for repairing urinary tract defects [39].

## 4. Stem Cell-Based Disease Modeling

The proliferation and differentiation capacity of human stem cells allows scientists to use them as more clinically relevant models to study the causes, pathologies, and mechanisms of certain diseases, and further develop rational therapeutic strategies. So far stem cells have been widely used in modeling many diseases resulting from genetic risks, environmental stressors (e.g., drugs, virus infection, and high glucose), injury, and aging. 

The availability ESCs and the advent of patient-specific iPSCs have opened new opportunities to investigate gene mutation and genetic risk variants in living disease-relevant cells [38] such as long QT syndrome, amyotrophic lateral sclerosis (ALS), and genetic variants-associated psychiatric disorders. Long QT syndrome mutation carriers have higher risk of cardiac events than unaffected family members [53]. Shal et al. modeled long QT syndrome type-2 (LQT2) syndrome using patient-specific iPSC-CMs. Such iPSCs were derived from asymptomatic and symptomatic HERG (Human Ether-à-go-go-Related Gene) mutation carriers from the same family. They found differences at CM-aggregate levels in the phenotype in-vitro between iPSC-CMs from LQT2 asymptomatic and symptomatic individuals. They also found that iPSC-CM aggregates from symptomatic patients, compared to asymptomatic patients, were more arrhythmic on rapid (IKr) and slow (IKs) delayed rectifier potassium channel blocks. This data suggests that this in-vitro human iPSC-CM model recapitulates major phenotype characteristics observed in LQT2 mutation carriers [27].

ALS is a complex neurodegenerative disorder characterized by the loss of the upper and lower motor neurons. Approximately 10% of cases are caused by specific mutations in known genes, with the remaining cases having no known genetic link. As such, sporadic cases have been more difficult to model experimentally. Seminary et al. generated ALS iPSCs from discordant identical twins and differentiated these patient-specific iPSCs into motor neurons. Their data indicates that a unique model of sporadic ALS may provide key insights into disease pathology and highlight potential differences between sporadic and familial ALS. Additionally, the iPSC lines from discordant identical twins generated in this study will be a valuable tool for studying sporadic ALS in an isogenic manner in order to better understand the phenotypes and pathological mechanisms in ALS [29].

Genome-wide association studies have identified an increasing number of genetic variants that significantly associate with psychiatric disorders. Hoffmann et al. analyzed the causal relationships between genetic risk variants and neuronal phenotypes, especially in schizophrenia and bipolar disorders. The authors also discussed the pros and cons of past and new research avenues, potential caveats, and upcoming developments in the field of ESC/iPSC-based modeling of causality in psychiatric disorders. Combinations of different approaches from iPSC-based modeling, animal studies, and deep patient phenotyping will enable stepwise progress on our understanding of psychiatric disorders and light up new perspectives on future therapies [38].

Stem cells are also widely used for modeling development and aging-related disorders. Claus et al. review the applications of human stem cells in modeling virus infections during early pregnancy. Several viruses such as the rubella virus, human cytomegalovirus, and Zika virus have been identified to be human teratogens. The underlying mechanisms of how such viral infections lead to abnormal cellular phenotypes and developments remain largely unknown. However, with the recent progress in pluripotent stem cell-based models including organoids and embryoids, it is now possible to assess congenital virus infections on a mechanistic level. The authors review the current status of using stem cell models for studying how congenital virus infections influence human cell and tissue development. The combination of congenital virology with the continuous advancements of stem cell-based technology will complement developmental biology and extend our current way of getting information on the function of genes active in human development [37]. For instance, iPSC-derived 3D cerebral organoids provide novel tools for studying biological mechanisms of neurological disorders. Song et al. discussed the cellular interactions and the physiological roles of neural cells with other cell types including endothelial cells and microglia based on iPSC models [42]. Yan et al. further described recent advances in modeling AD pathology and progression based on iPSC-derived brain organoids. These organoid systems, in combination with engineering tools, allow in vitro generation of brain-like tissues that recapitulate complex cell-cell and cell-extracellular matrix (ECM) interactions. The authored also discuss the influences of ECMs on the progression of neurodegeneration of AD [33].

As described in the Introduction, cancer stem cells are a subpopulation of cells that has the driving force of carcinogenesis. The development of strategies targeting cancer stem cells via drug transporters, specific surface markers, inhibiting signaling pathways or their components, and destroying their tumor microenvironment have multifocal effects that may improve the clinical outcome of patients with cancer. Urothelial cell carcinoma of the bladder is considered as a stem cell disease. Abugomaa et al. discussed the various types of bladder cancer stem cell heterogeneity, important regulatory pathways of cancer stem cells, and their roles in tumor progression and tumorigenesis, and the related experimental culture models. They also described the current stem cell-based therapies for bladder cancer disease. Patient-derived bladder cancer stem cell-included organoids could represent a faithful model system for studying tumor evolution and treatment response in context of precision cancer medicine [10].

## 5. Conclusions

Collectively, stem cell science is one of fastest moving fields of research. Each improvement in stem cell isolation, cell differentiation, reprogramming technology, and CRISPR/Cas9 genome engineering will extend the utility of stem cells further. This Special Issue presents state-of-the-art research of various types of stem cells including adult stem cells, iPSCs, ESCs, and cancer stem cells, alongside stem-cell derived organoids. This volume highlights the most recent developments and applications of stem cells in basic science research and disease treatments, alongside current limitations and future direction. 
